# Association of Urinary Tract Infection in Women with Bacterial Vaginosis

**DOI:** 10.4103/0974-777X.56254

**Published:** 2009

**Authors:** A H Sumati, N K Saritha

**Affiliations:** *Department of Microbiology, J. N. Medical College, Belgaum, Karnataka, India*; 1 *Department of Microbiology, V. M. Medical College, Sholapur, Maharashtra, India*

Sir,

Bacterial vaginosis is the most common vaginal infection. Its prevalence varies from 10% to 65% and it is mainly associated with sexually transmitted diseases. Many serious obstetric and gynecological complications have been associated with bacterial vaginosis. Obstetric complications include preterm labor and delivery, premature rupture of membranes, chorioamnionitis and endometritis.[1] Urinary tract infections are very common, which result in more than 7 million outpatient visits each year[2]; and two thirds of the patients are women.[3] The most important complications of urinary tract infection are pyelonephritis, sepsis and premature delivery. Treatment of women with urinary tract infections also needs treatment of genital tract infections. In 1989 the relationship between bacterial vaginosis and urinary tract infections in women using diaphragms was reported.[4] In 2000 there was a report that women suffering from bacterial vaginosis (BV) are at a greater risk of urinary tract infections than others.[5] In 2002 the same investigators reported the association of urinary tract infections in pregnant women with bacterial vaginosis. Considering the presence of only few research studies and lack of information about the incidence of this relationship and availability of fast and cost-effective diagnostic methods, we have carried out this study.[5]

A total of 174 women (82 pregnant and 92 nonpregnant) with complaint of vaginal discharge and attending Obstetrics and Gynecology clinics were selected for this study. From each patient, informed consent to participate in the study was obtained after explaining the study protocol. Detailed clinical history and demographical features were recorded. Both vaginal swab specimens and midstream urine specimens were collected from each patient. Women who had any type of sexually transmitted disease and other vaginal infections; and women on treatment for vaginal infections, diaphragms, douches and spermicides were excluded from the study. Each woman had pelvic examination. The pH of the discharge sample from lateral walls of vagina was assessed, and then a saline wet mount and whiff test (amine) with 10% potassium hydroxide solution were performed. The diagnosis of bacterial vaginosis was made when 3 of 4 Amsel’s criteria were present: homogenous white adherent vaginal discharge, vaginal pH >4.5, fishy amine odor from vaginal fluid when mixed with 10% potassium hydroxide and presence of clue cells in at least 20% of epithelial cells on a saline wet mount. Urinary tract infection was diagnosed by growth of at least 100,000 colony-forming units of a urinary pathogen per milliliter in culture of a midstream urine sample. Specimens containing high colony counts with more than one species of bacteria in asymptomatic women were considered contamination. All infections were then treated accordingly with appropriate antibiotics. The association (risk) of urinary tract infection was assessed by calculating the odds ratio with 95% confidence interval.

A total of 119 (68.39%) of the 174 women had bacterial vaginosis, whereas 58 women showed presence of urinary tract infection. Out of the 119 BV-positive women, 55 (46.21%) were pregnant and 64 (53.78%) were nonpregnant. Out of the 55 BV-positive pregnant women, 26 (42.27%) had urinary tract infection and 29 (52.72%) did not. Of the 64 BV-positive nonpregnant women, only 32 (50%) had urinary tract infection (OR, 13.75) [Table 1]. Once the difference in pregnancy status was taken into account, women with BV had odds of experiencing urinary tract infection that were 13.75 times more when compared with women who did not have BV. Our study showed that women with BV have significantly increased risk of urinary tract infections, with an odds ratio of 13.75.

**Table 1:** Prevalence of urinary tract infections in bacterial vaginosis

	BV	NBV	OR
Pregnant women			
UTI+VE	26	3	7.17
UTI−VE	29	24	
Nonpregnant women			
UTI+VE	32	0	53.3
UTI−VE	32	28	

Mantel-Haenszel odds ratio = 13.75

Hillerbrand *et al.*[5] in a cross-sectional study examined 503 pregnant women from the viewpoint of urinary tract infections and BV who came for initial prenatal visit and reported that 13.6% of the 140 women suffering from BV also had urinary tract infection (UTI); whereas only 6.6% of the 363 women without BV had UTI. They concluded that BV in pregnancy increases the risk of UTI. Maryam Afrakhteh *et al.*[6] in a case-control study examined 67 patients with UTI and compared them with 67 normal individuals. BV was reported in 40.3% and 62.7% in the control group and study group, respectively.

Association of BV with UTI (vice versa) probably begins with an increase in the pH of the vagina because of reduction of vaginal lactobacilli-producing lactate and hydrogen peroxide. The normal vaginal flora may be replaced by predominantly anaerobic flora.[5] Frequent sexual intercourse, which was also linked to both these infections, may also contribute to this phenomenon.[1] Factors causing colonization of gram-negative bacilli around urethra are unknown, but it seems that urethral massage during sexual activity has a facilitating role; furthermore, it seems that proximity of urethra to anus, shortness of female urethra, its location under labia, warm and moist environment of perineum have important roles to play. It might be necessary to carry out test for urinary tract infections in women with bacterial vaginosis (vice versa). Evaluating for urinary tract infections in women with bacterial vaginosis is cost effective, in that it might reduce the risk of associated complications. Clinical management in pregnant women might be more complicated because of potential perinatal problems associated with those infections.[1] Further studies are needed to evaluate the exact sequence of events.
